# Effects of SARS-CoV-2 Vaccines on Sperm Quality: Systematic Review

**DOI:** 10.2196/48511

**Published:** 2023-12-06

**Authors:** Guanjian Li, Rongqiu Zhang, Bing Song, Chao Wang, Qunshan Shen, Xiaojin He, Yunxia Cao

**Affiliations:** 1 Reproductive Medicine Center, Department of Obstetrics and Gynecology, the First Affiliated Hospital of Anhui Medical University Hefei China; 2 National Health Commission Key Laboratory of Study on Abnormal Gametes and Reproductive Tract Hefei China; 3 Reproductive Medicine Center, the Affiliated Jinyang Hospital of Guizhou Medical University Guiyang China; 4 The Second People's Hospital of Guiyang Guiyang China; 5 Reproductive Medicine Center, Human Sperm Bank, Department of Obstetrics and Gynecology, the First Affiliated Hospital of Anhui Medical University Hefei China; 6 Key Laboratory of Population Health Across Life Cycle Ministry of Education of the People’s Republic of China Hefei China; 7 Reproductive Medicine Center, Department of Obstetrics and Gynecology, Shanghai General Hospital, Shanghai Jiao Tong University School of Medicine Shanghai China

**Keywords:** COVID-19 vaccine, SARS-CoV-2, reproductive system, fertility, sperm quality

## Abstract

**Background:**

The COVID-19 pandemic, caused by SARS-CoV-2, has triggered a global public health crisis of unprecedented proportions. SARS-CoV-2 vaccination is a highly effective strategy for preventing infections and severe COVID-19 outcomes. Although several studies have concluded that COVID-19 vaccines are unlikely to affect fertility, concerns have arisen regarding adverse events, including the potential impact on fertility; these concerns are plagued by limited and inconsistent evidence.

**Objective:**

This review aims to provide a recent assessment of the literature on the impact of COVID-19 vaccines on male sperm quality. The possible impact of COVID-19 vaccines on fertility potential was also examined to draw a clearer picture and to evaluate the effects of COVID-19 on male reproductive health.

**Methods:**

PubMed, Scopus, Web of Science, Embase, and Cochrane databases were searched from their inception to October 2023. Eligible studies included articles reporting SARS-CoV-2 vaccination and human semen quality and fertility, as well as the impact of vaccination on assisted reproductive technology treatment outcomes. The quality of cohort studies was assessed using the Newcastle-Ottawa Scale, and the quality of cross-sectional studies was assessed using the quality evaluation criteria recommended by the Agency for Healthcare Research and Quality. The systematic review followed PRISMA (Preferred Reporting Items for Systematic Reviews and Meta-Analyses) guidelines.

**Results:**

The initial literature search yielded 4691 records by searching 5 peer-reviewed databases (PubMed, Scopus, Web of Science, Embase, and Cochrane). Finally, 24 relevant studies were selected for our study. There were evident research inequalities at the regional level, with the United States and Western European countries contributing 38% (9/24) of the studies, Middle Eastern countries contributing 38% (9/24), China accounting for 21% (5/24), and Africa and South America accounting for none. Nonetheless, the overall quality of the included studies was generally good. Our results demonstrated that serious side effects of the COVID-19 vaccine are extremely rare, and men experience few problems with sperm parameters or reproductive potential after vaccination.

**Conclusions:**

On the basis of the studies published so far, the COVID-19 vaccine is safe for male reproductive health. Obviously, vaccination is a wise option rather than experience serious adverse symptoms of viral infections. These instances of evidence may help reduce vaccine hesitancy and increase vaccination coverage, particularly among reproductive-age couples. As new controlled trials and prospective cohort studies with larger sample sizes emerge, the possibility of a negative effect of the COVID-19 vaccine on sperm quality must be further clarified.

## Introduction

### Background

The COVID-19 pandemic, caused by SARS-CoV-2, has severely affected global public health, economics, and social life since 2019 [1-3]. To establish herd immunity and effectively reduce the risk of COVID-19 infection, the medical and research community conducted many research studies within a short period to develop the SARS-CoV-2 vaccines. Impressively, these efforts have ultimately led to the development of multiple efficacious vaccines in a short period, which is an unprecedented and important accomplishment [4]. Vaccination is proven to be an effective tool for reducing the risk of infection and severe COVID-19 outcomes and is now a global priority [5,6].

Earlier reports have confirmed that the testis is one of the tissues with the highest expression of angiotensin-converting enzyme 2 messenger RNA (mRNA) and protein, both of which are key targets for SARS-CoV-2 to enter host cells [7]. The inflammatory state caused by COVID-19 may undermine the integrity of the blood-testis barrier and further promote the infiltration of the virus. Therefore, in the early stages of the COVID-19 epidemic, some researchers argued that the testes might be susceptible to SARS-CoV-2. In fact, a series of studies have confirmed a short-term reduction in testosterone production and semen quality in patients with COVID-19. Semen parameters, including semen volume, sperm concentration, total sperm count, total sperm motility, and progressive sperm motility, were negatively affected by SARS-CoV-2 infection [8,9]. On the basis of the aforementioned potential damage to the reproductive system following SARS-CoV-2 infection, some researchers suspected that vaccines that mimic the virus could also affect fertility via a similar mechanism. Numerous types of disinformation and misinformation have emerged on social media, including questioning the relationship between COVID-19 vaccination and male fertility [10-12]. This public fear is partly owing to a lack of understanding of the newly developed COVID-19 vaccine [13,14].

It is also important to note that the current authoritative evidence on the impact of COVID-19 vaccines on male fertility is still limited, and the results are controversial. The lack of evidence for a definitive conclusion could discourage the public, fuel concerns about the potential impact of the vaccine on male fertility, and lead to vaccine hesitancy [15-17].

### Objectives

As the pandemic continues to evolve, the number of newly approved vaccines and scientific studies will continue to increase [18]. In this study, we systematically reviewed the latest data and theoretical considerations on the impact of COVID-19 vaccines on semen quality in men to provide more conclusive information for policy makers, health care providers, media, and the public.

## Methods

### Search Strategy and Selection Criteria

According to the PRISMA (Preferred Reporting Items for Systematic Reviews and Meta-Analyses) guidelines, we conducted a systematic review of empirical articles on SARS-CoV-2 vaccination, human semen quality and fertility, and the impact of vaccination on assisted reproductive technology (ART) treatment outcomes. The PRISMA checklist is provided in Multimedia Appendix 1. The PubMed, Scopus, Web of Science, Embase, and Cochrane databases were used to identify all relevant literature published up to October 2023. A keyword search strategy was developed with the following terms: “COVID-19,” “COVID19,” “covid19,” “covid-19,” “coronavirus,” “novel coronavirus,” “new coronavirus,” “SARS Coronavirus 2 Infection,” “2019 Novel Coronavirus Disease,” “2019 Novel Coronavirus Infection,” “SARS-CoV-2,” “severe acute respiratory syndrome coronavirus 2,” “COVID-19 Virus Disease,” “COVID-19 Virus Infection,” “Coronavirus Disease-19,” “Vaccines,” “vaccine,” “vaccination,” “COVID-19 Vaccines,” “COVID-19 Vaccines/adverse effects,” “SARS-CoV-2 Vaccine,” “Coronavirus Disease 2019 Vaccine,” “2019-nCoV Vaccine,” “SARS Coronavirus 2 Vaccine,” “BNT162b2 mRNA vaccine,” “COVID-19 mRNA Vaccine,” “Fertility,” “Fecundability,” “Fecundity,” “Infertility, Male,” “Infertility, Female,” “sterility,” “IVF,” “Fertilization in Vitro,” “reproductive function,” “ART,” “sperm,” “spermatozoa,” “semen analysis,” “spermatogenesis,” and “semen parameter.” The search strategies are provided in detail in Multimedia Appendix 2.

**Figure 1:**
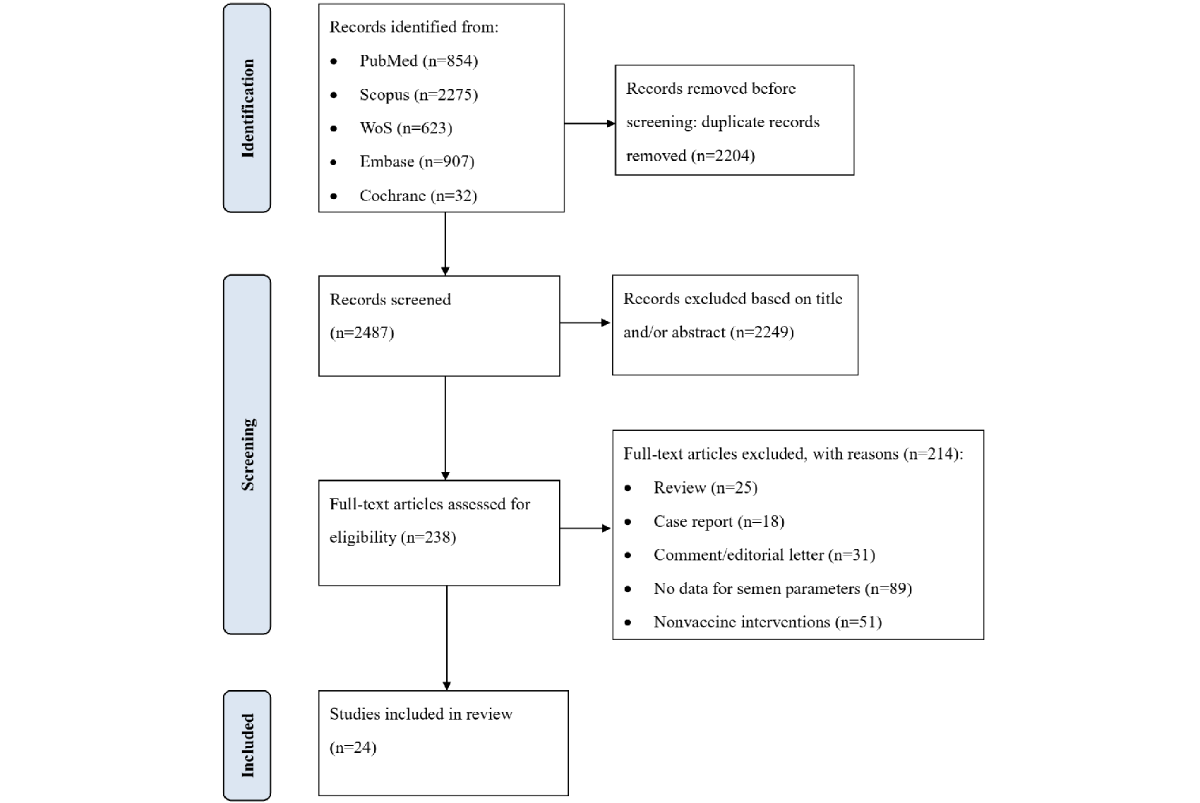
The PRISMA (Preferred Reporting Items for Systematic Reviews and Meta-Analyses) flowchart of the screening and selection process. WoS: Web of Science.

The search was restricted to articles on humans, published in any language, without additional restrictions. With regard to publication type, original studies were selected. Abstracts, comments, reviews (narrative or systematic), modeling only, case reports, case series (reporting data for <10 patients), and editorials were excluded. We evaluated the relevance and quality of the text. Two independent reviewers (GL and BS) screened all the studies by title, abstract, and keywords to identify potentially relevant articles. All articles obtained through the search strategy were imported into Rayyan (Qatar Computing Research Institute), and duplicates were removed. Papers that did not meet the inclusion criteria for the full-text evaluation were excluded. Subsequently, the full texts were screened to collect the data. Furthermore, the reference lists of the included studies were manually searched to identify additional eligible studies and to ensure that relevant studies were not missed. Eligible studies that were chosen to be included in this study were those evaluating the impacts of the COVID-19 vaccine on semen quality and male reproduction. Articles related to female vaccination and those without information on male vaccination were excluded. Data on the effect of the SARS-CoV-2 on male fertility were also excluded. The inclusion and exclusion criteria are presented in Table 1. Any disagreements were resolved through discussion among the study team members. The flow and results of the literature search are summarized in a PRISMA flowchart (Figure 1). Ethics approval was not required for this study because the data were derived from previously published studies.

The initial literature search yielded 4691 records. Of these, 2204 duplicate articles were removed, 2249 records were further excluded based on title or abstract screening, and 214 were excluded based on full-text assessment. Finally, 24 studies were selected for evaluation. We extracted the data using a pretested data extraction form created in Microsoft Excel 2019 (Microsoft Corporation). Article information (eg, PubMed Identifier, first author, year of publication, and country); study design; vaccine type; side effects; and changes in semen parameters were extracted from the articles.

**Table 1:** Summary of the inclusion and exclusion criteria.

Parameter	Inclusion criteria	Exclusion criteria
Article or study type	Population-based original research studies	Abstracts, comments, and editorials Reviews and book chapters Protocols and modeling only Case reports and case series Animal experiment
Language	Any language	Without additional restrictions
Publication period	November 1, 2019, to October 1, 2023	All dates outside the span from November 2019 to October 2023
Study content	Studies assessing the impact of COVID-19 vaccines on semen quality and male reproduction	Studies related to female vaccination without information on male vaccination Data on the effects of COVID-19 on male fertility

### Characteristics of the Included Literature

The methodological quality of the included studies was evaluated according to study design. Cohort studies were assessed using the Newcastle-Ottawa Scale, which provides a score between 0 and 9 and assesses the following quality parameters: comparability of study groups, selection of study groups, and ascertainment of outcomes [19]. Cross-sectional studies were evaluated using 11 entries from the quality evaluation criteria recommended by the Agency for Healthcare Research and Quality [20]. Each entry contained options “Yes,” “No,” and “Unclear.” “Yes” was rated as 1 point, while “No” and “Unclear” were counted as 0. Scores of 0 to 3, 4 to 7, and 8 to 11 were considered as low-, medium- and high-quality studies, respectively.

## Results

### COVID-19 Vaccine Platform

**Figure 2:**
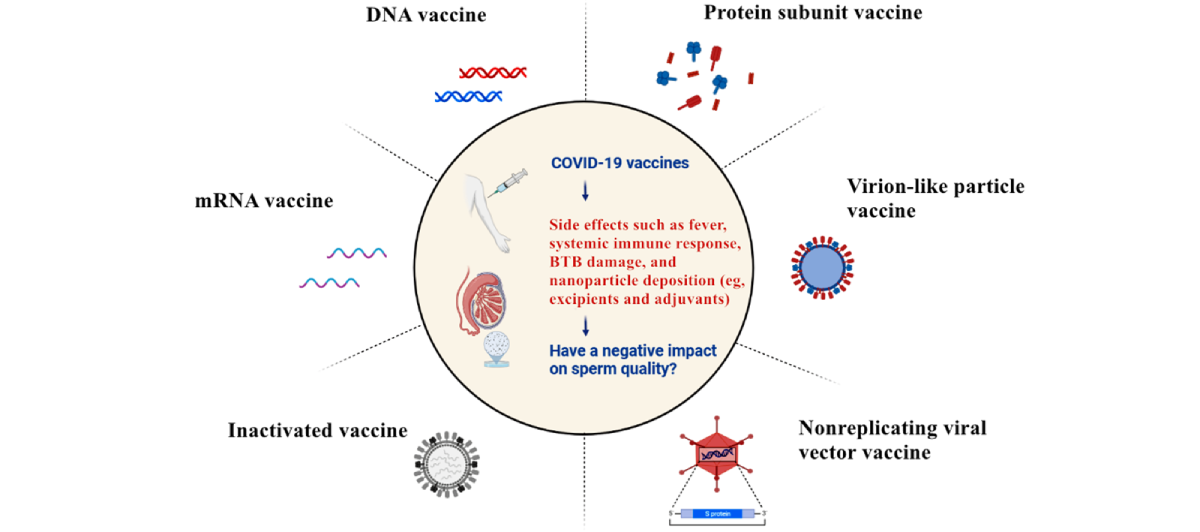
Different platform types of COVID-19 vaccines and the possible mechanisms affecting semen quality. BTB: blood-testis barrier; mRNA: messenger RNA.

Vaccines are critical and cost-effective tools to control the COVID-19 pandemic. Since the onset of the COVID-19 pandemic, research groups from various countries have made significant progress in developing vaccines in a short period using a variety of platform technologies, with >100 vaccine candidates currently undergoing preclinical development worldwide [21]. These vaccine platforms range from protein-based (eg, subunits and virus-like particles) and virus-based (eg, attenuated live and inactivated vaccines) strategies to gene-based strategies [22,23]. The different types of vaccine platforms are shown in Figure 2. In this study, we focused on several widely used vaccines that have been reported to affect semen quality.

### mRNA Vaccines

Several large biotechnology companies such as Pfizer, BioNTech, and Moderna have developed COVID-19 vaccines using advanced mRNA vaccine platforms. In the United States, the Food and Drug Administration approved emergency authorizations for the BNT162b2 (Pfizer-BioNTech) and mRNA‐1273 (Moderna) vaccines in December 2020 [24-26]. mRNA vaccines involve an innovative approach, borrowing the cell’s transcriptional machinery to produce the desired set of particles. Vaccine mRNA can enter cells and can be converted into SARS-CoV-2 glycoproteins (spike proteins). The spike proteins elicit an immune response by activating helper T and B cells, eventually establishing immunity against SARS-CoV-2 [27]. These mRNA-based vaccines are noninfectious. They do not contain any live viral particles and are synthesized by in vitro transcription in the absence of microbial molecules.

### Recombinant Viral-Vectored Vaccines

Recombinant viral-vectored vaccines use a bioengineered viral vector that can express and clone antigens from the target pathogen [28]. Lentiviruses, retroviruses, adenoviruses, and adeno-associated viruses are the common vectors used in this platform. Nonreplicating viral vaccines have been extensively studied and have a well-established track record for preventing infectious diseases because of their genetic malleability, safety, and ability to stimulate strong cellular immune responses without the use of adjuvants. Furthermore, a single dose of viral vector–based vaccines may provide adequate protection. Several COVID-19 vaccines based on recombinant viral vector technology including Sputnik V (Gamaleya Institute), ChAdOx1 (Oxford Astra Zeneca), and Ad26. COV2. S (Johnson & Johnson Janssen) have been approved for emergency use worldwide [29].

### Inactivated Vaccines

Chemically or physically inactivated viral vaccines are among the oldest vaccine design approaches and have been used successfully against a variety of human viral infections such as hepatitis A, polio, and influenza [30]. This method involves injecting an inactivated virus into the host, inducing an immune response and promoting strong immunity against the virus. In contrast to their live attenuated counterparts, inactivated viral vaccines pose few safety concerns and deliver a wide range of native viral antigens, including surface antigens with conserved epitope conformations, which can stimulate conformation-dependent antibody responses. As completely inactive viruses do not replicate, repeated administration and adjuvants are required to stimulate the immune system and ensure that these vaccines work properly [31]. Currently, several vaccines, including CoronaVac (Sinovac) and BBIBP-CorV (Sinopharm), are being widely used in several countries, including China.

### Effects of SARS-CoV-2 Vaccination on Semen Quality: Available Evidence

All available COVID-19 vaccines had acceptable efficacy and safety profiles in phase 3 clinical trials [32]. As of January 2023, >13 billion doses of the vaccine had been administered worldwide, with an excellent safety and efficacy profile. However, as reproductive toxicity has not been evaluated in clinical trials, one reason for vaccine hesitancy is its potential negative effects on fertility [33].

As shown in Table 2, we identified 24 studies that evaluated the effects of different COVID-19 vaccines on semen quality (some studies simultaneously evaluated >1 vaccine). All the studies were published between March 2020 and October 2023. The overall quality of both the cross-sectional and cohort studies is shown in Table 2.

All the studies were of reasonable quality. Of 24 studies, 9 (38%) studies were conducted in the United States and Western European countries [34-42], 5 (21%) in China [43-47], 1 (4%) in Indonesia [48], and 9 (38%) in the Middle Eastern countries [49-57]. In total, 3 (12%) studies used inactivated COVID-19 vaccines, 4 (17%) studies used viral-vectored vaccines, 8 (33%) studies used mRNA vaccines, and 3 (12%) studies included a combined analysis of multiple types of vaccines. A total of 11 (46%) studies involved patients with infertility undergoing ART, 11 (46%) involved healthy volunteers or sperm donors, and 2 (8%) involved other male populations with type 2 diabetes and nonserious chronic conditions. All studies described standard methods for sample collection and processing and followed the fifth edition of the World Health Organization laboratory manual. ’The study design, study population, vaccine type, result analysis, and adverse reaction reports of all 24 studies are listed in Table 3. These studies reported a variety of semen parameters: 9 (38%) reported total sperm count, 23 (96%) reported sperm concentration, 7 (29%) reported sperm morphology, 19 (79%) reported total sperm motility, 17 (71%) reported progressive sperm motility, and 11 (46%) reported total motile sperm count (TMSC).

**Table 2:** Summary of the inclusion and exclusion criteria.

Source	Country	Study design	AHRQ^a^ or NOS^b^ score	Interpretation
Diaz et al [34], 2021-2022	United States	Prospective cross-sectional	6	Medium quality
Gonzalez et al [35], 2020-2021	United States	Prospective cross-sectional	5	Medium quality
Wang et al [43], 2021	China	Prospective cross-sectional	6	Medium quality
Huang et al [44], 2021-2022	China	Retrospective cross-sectional	8	High quality
Zhu et al [45], 2020-2021	China	Retrospective cross-sectional	8	High quality
Xia et al [46], 2021	China	Retrospective cohort study	7	High quality
Dong et al [47], 2023	China	Retrospective cohort study	8	High quality
Abd et al [51], 2022	Iraq	Prospective cross-sectional	4	Medium quality
Al-Alami et al [50], 2021	Jordan	Retrospective cohort study	5	Medium quality
Alenzi et al [49], 2021	Saudi Arabia	Prospective cohort study	5	Medium quality
Lestari et al [48], 2020-2022	Indonesia	Prospective cohort study	8	Medium quality
Karavani et al [52], 2021-2022	Israel	Retrospective cross-sectional	7	Medium quality
Gat et al [53], 2021-2022	Israel	Retrospective cross-sectional	7	Medium quality
Lifshitz et al [54], 2021	Israel	Prospective cross-sectional	5	Medium quality
Safrai et al [55], 2021	Israel	Retrospective cross-sectional	9	High quality
Barda et al [56], 2020-2021	Israel	Prospective cross-sectional	4	Medium quality
Orvieto et al [57], 2021	Israel	Retrospective cohort study	6	Medium quality
Olana et al [36], 2021	Italy	Prospective cross-sectional	8	High quality
Reschini et al [37], 2022	Italy	Retrospective cross-sectional	5	Medium quality
Massarotti et al [38], 2021	Italy	Prospective cross-sectional	6	Medium quality
Chillon et al [42], 2021	Germany	Retrospective cross-sectional	7	Medium quality
Drapkina et al [39], 2021	Russia	Prospective cross-sectional	7	Medium quality
Esaulenko et al [40], 2021	Russia	Prospective cross-sectional	7	Medium quality
Elagin et al [41], 2021-2022	Russia	Prospective cross-sectional	5	Medium quality

^a^AHRQ: Agency for Healthcare Research and Quality.

^b^NOS: Newcastle-Ottawa Scale.

**Table 3:** The effect of COVID-19 vaccines on semen parameters.

Source	Country	Vaccine type	Sample size; population	Age (y)	Semen test before vaccination	Semen test after vaccination, days	Outcomes	Side effects
Diaz et al [34], 2021-2022	United States	58% Moderna vaccines and 42% Pfizer vaccines	12; normal volunteers	Mean 26; range 25-30	Before the first dose of vaccine	T1^a^: 3 months after the first dose and T2^b^: at least 9 months after the second dose	No significant differences	Not reported
Gonzalez et al [35], 2020-2021	United States	21 received BNT162b2 and 24 received mRNA-1273	45; normal volunteers	Mean 28; range 25-31	Before the first dose of vaccine	75 (IQR 70-86) days after the second dose	After vaccination, the median sperm concentration, median TMSC^c^, semen volume, and sperm motility were significantly increased	Not reported
Wang et al [43], 2021	China	Inactivated vaccine: CoronaVac (Sinovac)	153; patients with infertility undergoing IVF^d^ treatment	Mean 33; range 29-37	Before the first dose of vaccine	Median 71 (IQR 38-102) days after the second dose	After vaccination, the amount of semen increased, the vitality decreased, and the normal morphology of sperm decreased	Not reported
Huang et al [44], 2021-2022	China	Inactivated vaccine: BBIBP-CorV (Sinopharm) or CoronaVac (Sinovac)	128; men with previous semen examination in the medical center	Mean 31; range 29-35	Before the first dose of vaccine	Median 87.5 (IQR 52.0-137.5) days after the second dose	No significant differences	Not reported
Zhu et al [45] (2020-2021)	China	Inactivated vaccine	43; semen donors from sperm bank	Mean 28.6 (SD 5.9)	Before the first dose of vaccine	T1: mean 9.1 (SD 8.1) days after the first dose and T2: mean 30.1 (SD 23.3) days after the second dose	No significant differences	A sore arm for both the first and second injections (nearly 13.9% and 4.7%, respectively). One donor reported feeling tired
Xia et al [46], 2021	China	Inactivated vaccine: BBIBP-CorV (Sinopharm) or CoronaVac (Sinovac)	Vaccine group (n=105) and control group (n=155); patients undergoing IVF treatment	Vaccine group: mean 33.9 (SD 4.7) and control group: mean 33.3 (SD 4.4)	Not tested	Unvaccinated group and vaccinated group: average 80.6 days after the first dose	No significant differences	Pain at the injection site (11.43%), followed by fatigue (6.67%), headache (1.90%), nausea (0.95%), and low-grade fever (0.95%)
Dong et al [47], 2023	China	Inactivated vaccine	519; fertile men	Mean 35.3 (SD 4.3)	Not tested	Unvaccinated group and vaccinated group: ≤90 days or >90 days after the first dose	No significant differences	The main adverse effect was injection site pain or redness; percentage of participants with fever (first dose: 0.3%, second dose: 0.6%, and booster dose: 1.9%)
Abd et al [51], 2022	Iraq	Pfizer-BioNTech (BNT162b2)	60; men undergoing IVF treatment (infertility due to clear female factors)	Mean 37.2 (SD 5.3)	Before the first dose of vaccine	Mean 101 (SD 37) days after the first dose	Both total motility and progressive motility were slightly reduced after vaccination	Not reported
Al-Alami et al [50], 2021	Jordan	Pfizer (44.1%), Sinopharm (31.4%), Astra Zeneca (7.9%), and Moderna (0.3%)	354; participants who visited 1 infertility unit	Mean 35.6 (SD 7.9)	Not tested	Unvaccinated group and vaccinated group: clear data were not available	The sperm concentration was higher among vaccinated participants than unvaccinated participants	Not reported
Alenzi et al [49], 2021	Saudi Arabia	Pfizer-BioNTech (44%), Oxford and Astra Zeneca (32%), and mixed (24%)	100; normal volunteers	Mean 37.89 (SD 10.3)	Before the first dose of vaccine	1 month after the second dose	There was a significant increase in progressive sperm motility: prevaccination motility: median 55.03 (IQR 42-61.75) vs postvaccination motility: median 56.5 (IQR 42.25-63)	Not reported
Lestari et al [48], 2020-2022	Indonesia	CoronaVac (Sinovac), Astra Zeneca, and Moderna: proportion is not clear	70; patients with infertility	Vaccine group: mean 29.8 (SD 3.5) and control group: mean 29.3 (SD 4.5)	Before the first dose of vaccine	2 to 4 weeks after the second dose	The viral-vector vaccine caused a decrease in morphology as well as an increase in DFI^e^	Not reported
Karavani et al [52], 2021-2022	Israel	Pfizer-BioNTech (BNT162b2)	58; men undergoing IVF treatment	Mean 38.0 (SD 6.1)	Before the first dose of vaccine	6 to 14 months after vaccination	No significant differences	Not reported
Gat et al [53], 2021-2022	Israel	Pfizer-BioNTech (BNT162b2)	37; semen donors from sperm banks	Mean 26.1 (SD 4.2)	Before the first dose of vaccine	T1, T2, and T3^f^: included 1 to 3 semen samples per donor who provided the samples 15 to 45 days, 75 to 125 days, and >145 days after vaccination	Compared with sperm concentration at T0^g^, the concentration at T2 decreased by 15%, and total motor count decreased by 22%. Subsequently, T3 evaluation showed that the damage had recovered	Not reported
Lifshitz et al [54], 2021	Israel	Pfizer-BioNTech (BNT162b2)	75; normal volunteers, 40% of whom were medical staff	Mean 38.6 (SD 4.3)	Not tested	On average, 37 days after the second dose	Compared with the WHO^h^ reference range, there are only 2 borderline sperm specimens	Fatigue (34.7%), pain at the injection site (13.3%), fever (9.3%), and chills (8%)
Safrai et al [55], 2021	Israel	Pfizer-BioNTech (BNT162b2)	72; patients undergoing IVF treatment	Mean 35.7; range 33-43	Before the first dose of vaccine	Median 71.0 (IQR 40.5-104.8) days after the first dose	No significant differences	Not reported
Barda et al [56], 2020-2021	Israel	Pfizer-BioNTech (BNT162b2)	33; semen donors from sperm banks	27	Before the first dose of vaccine	The average sperm parameters of multiple samples were used for comparison. For each donor, at least 1 sample was received ≥72 days after the second dose	Total sperm count and TMSC increased after the second dose of vaccine	Pain at injection site: 79% (T1) and 88% (T2) of participants, lethargy: 9% (T1) and 48% (T2) of participants, and fever: 1 case (T1)
Orvieto et al [57], 2021	Israel	Pfizer-BioNTech (BNT162b2)	36; patients with infertility undergoing IVF treatment	Mean 40.1 (SD 4.8)	Before the first dose of vaccine	Mean 33.3 (SD 14.9) days after the second dose	No significant differences	Not reported
Olana et al [36], 2021	Italy	Pfizer-BioNTech (BNT162b2)	47; normal volunteers	Mean 29.3 (SD 6.0)	Before the first dose of vaccine	70 days from the second dose	No significant differences	Not reported
Reschini et al [37], 2022	Italy	Pfizer-BioNTech (69%), Moderna (19%), Oxford and Astra Zeneca (9%), Johnson & Johnson’s Janssen (1%), and mixed (2%)	106; fertile men undergoing ART^i^ programs	Mean 39; range 36-42	Before the first dose of vaccine	Median 75 (IQR 38-112) days after the first dose	No significant differences	45% of the patients reported mild, self-resolving adverse events after the vaccine, including pain at injection site, fever, fatigue, nausea, muscle pain, and diarrhea
Massarotti et al [38], 2021	Italy	76% received mRNA vaccines, 20% viral-vector vaccines, 2% a mixed formulation, and 2% were not clear about the type	101; men undergoing fertility treatments	Mean 37.5 (SD 5.5)	Before the first dose of vaccine	Mean 2.3 (SD 1.5) months after the second dose	After vaccination, the median volume of the sample decreased from 3.0 to 2.6 ml. Sperm concentration, progressive motility, and TMSC increased	Not reported
Chillon et al [42], 2021	Germany	Pfizer-BioNTech (not reported)	86; normal volunteers	Vaccine group: mean 38 (SD 5.5) and control group: mean 36 (SD 6.5)	Not tested	Unvaccinated group and vaccinated group: clear data were not available	SARS-CoV-2 vaccination parameters and vaccine-induced antibodies were not associated with sperm parameters	Not reported
Drapkina et al [39], 2021	Russia	Viral-vector vaccine: Sputnik V	45; normal volunteers	Mean 36; range 30-44	Before the first dose of vaccine	90 days after the first dose	No significant differences	Not reported
Esaulenko et al [40], 2021	Russia	Viral-vector vaccine: Sputnik V	30; patients with type 2 diabetes	Mean 46; range 42-48	Before the first dose of vaccine	90 days after the first dose	No significant differences	19 (63%) patients demonstrated a temperature rise and 26 (87%) patients reported tenderness at injections site
Elagin et al [41], 2021-2022	Russia	Viral-vector vaccine: (Sputnik V)	44; normal volunteers	Mean 22.4 (SD 4.7)	Before the first dose of vaccine	10-12 days after the first dose and 32-36 days after the second dose	No significant differences	A total of 5 and 7 participants exhibited fever with headache after the first and second vaccine doses, respectively

^a^T1: time point 1.

^b^T2: time point 2.

^c^TMSC: total motile sperm count.

^d^IVF: in vitro fertilization.

^e^DFI: DNA fragmentation index.

^f^T3: time point 3.

^g^T0: time point 0.

^h^WHO: World Health Organization.

^i^ART: assisted reproductive technology.

## Discussion

### Principal Findings

Safrai et al [55] published the first study on the impact of the COVID-19 vaccine on sperm parameters, demonstrating that the BNT162b2 mRNA vaccine (Pfizer or BioNTech) was not associated with a decrease in sperm quality. The study enrolled 72 individuals (57 of whom had normal sperm parameters). Sperm parameters in men with normal and abnormal sperm analysis did not change significantly after vaccination. The authors noted that after COVID-19 vaccination, none of the sperm parameters changed significantly [55]. In February and March 2021, Lifshitz et al [54] evaluated the effects of the Pfizer COVID-19 vaccine in 75 men with proven fertility [54]. The primary outcome was the percentage of abnormal sperm parameters in men 1 to 2 months after the second injection of the COVID-19 vaccine, including abnormal sperm morphology, reduced percentage of motile spermatozoa, and oligozoospermia rates. The results showed that the semen parameters after COVID-19 vaccination were mostly within the normal reference ranges established by the World Health Organization and did not indicate any causative negative effects of the COVID-19 vaccination. However, the participants in this study were a relatively homogeneous group, consisting of fertile male individuals from high socioeconomic groups. Moreover, participants were only followed up for 1 to 2 months after receiving the second dose of vaccine; therefore, long-term results have not yet been reported. In contrast, in a retrospective, longitudinal, multicenter study recently published in *Andrology*, the effects of the COVID-19 BNT162b2 vaccine on semen parameters were evaluated in 37 semen donors at different time points, before and after immunization [53]. Compared with the prevaccination levels, there was a selective decrease in sperm concentration and TMSC 75 to 125 days after vaccination (*P*=.01 and *P*=.007, respectively). Normal levels of these parameters were restored 145 days after vaccination, and no changes in sperm volume or motility were observed. Similarly, Abd et al [51] observed that the total motility and progressive sperm activity decreased significantly after BNT162b2 injection. Notably, considering that all semen parameters were still within the normal range, the investigator considered that the vaccine had no deleterious effects on semen parameters [51]. An additional prospective cohort study evaluated the effects of an inactivated vaccine (CoronaVac) on semen quality and in vitro fertilization (IVF) outcomes. A total of 542 patients undergoing IVF were divided into the unexposed group (nonvaccination) and the exposed group (vaccination), and it was found that the semen parameters seemed to fluctuate in the vaccinated men, including increased semen volume, decreased normal forms of sperm, and lower motility, whereas the motile sperm counts were similar. In addition, all semen parameters were above the lower reference limit. Therefore, researchers jointly believed that inactivated vaccines may not negatively affect sperm indicators or embryo development potential in men [43]. However, although this was a cohort study with a relatively large sample size, the male participants were all infertile, limiting the generalizability of the findings.

Surprisingly, significant improvements in some sperm parameters were noted after the COVID-19 vaccination. Massarotti et al [38] conducted a prospective study in which 101 vaccinated men (who were undergoing fertility treatments) had semen testing before vaccination and 2.3 (SD 1.5) months after the second dose of vaccine (76% of the participants were vaccinated with mRNA vaccine and 20% with viral-vectored vaccine). The investigators found a significant reduction in the median volume of the sample (from 3.0 to 2.6 mL), whereas sperm concentration, progressive motility, and TMSC increased (from 25.0 to 43.0 million/mL, from 50% to 56%, and from 34.8 to 54.6 million, respectively) [38]. In another prospective cohort study published in JAMA, Gonzalez et al [35] evaluated the effect of mRNA vaccines in 45 healthy volunteers (21 Pfizer and 24 Moderna) and found no significant decrease in sperm parameters. Semen samples were collected before the first dose and a median of 75 days after the second dose [35]. The baseline median sperm concentration was 26 million/mL, and the TMSC was 36 million. After the second vaccine dose, the median sperm concentration increased to 30 million/mL, and the median TMSC concentration increased to 44 million. Sperm motility and semen volume also increased significantly. The authors suggested that the increase in sperm parameters might be explained by known individual variations in sperm and increased abstinence time before postvaccine sample collection. Similarly, Barda et al [56] evaluated the effect of 2 doses of the vaccine (BNT162b2) on the sperm parameters of 33 sperm donors who donated sperm on multiple occasions. Compared with the percentage of motile sperm in the sample before vaccination, the percentage after the second dose of the vaccine did not change. However, the total sperm and motile counts increased unexpectedly.[56]. One possible explanation is that some participants changed their lifestyle habits or were more health conscious than the retrospectively enrolled infertile population, which acted as a confounding factor and positively affected sperm quality [58].

Two previous meta-analyses examined the effect of COVID-19 vaccination on sperm parameters based on published data [59,60]. Both studies suggested that the vaccination had no discernible negative impact on sperm quality. However, the 2 meta-analyses had nonnegligible limitations: the included studies used different types of vaccines, administered different doses of vaccines, had mostly unknown time intervals from vaccination to sperm analysis, had large differences in the participating populations, and had a small sample size for each group after the subgroup analysis. In other words, the methodological quality of meta-analyses assessing the impact of COVID-19 vaccination on semen parameters is currently unsatisfactory. As new controlled trials and prospective cohort studies with larger sample sizes emerge, the possibility of a negative effect of the COVID-19 vaccine on sperm quality must be further clarified.

Commonly reported side effects of the COVID-19 vaccine in premarketing clinical trials and postmarketing surveillance programs include systemic reactions (eg, fatigue, headache, and muscle pain) as well as injection site reactions (eg, pain, redness, and swelling), with rare serious adverse events [61,62]. Furthermore, fever is considered one of the most common side effects of vaccination [63]. According to the literature, fever can temporarily impair sperm parameters, causing a decline in sperm count, quality, and DNA integrity [64]. It should be noted that a range of studies have demonstrated fever to be a common adverse effect following multiple vaccine injections including mRNA vaccines (eg, BNT162b2), inactivated vaccines (eg, CoronaVac), and viral-vector vaccines (eg, Ad26. Sputnik V). Therefore, male patients who experience fever after receiving the COVID-19 vaccine may experience fluctuations in their sperm parameters. However, this effect is similar to that caused by fever due to other registered vaccines [41,47,54].

According to a study by Gat et al [53], the systemic immune response following BNT162b2 immunization may contribute to the deterioration of selective temporary sperm concentration and TMSC; however, the long-term prognosis remains favorable [53]. It is important to note that similar studies did not have data on the detailed health and immune status of the participants before vaccination. Therefore, their conclusions should be interpreted with caution. Carto et al [65] examined data from a large US electronic health record database on the risk of orchitis, epididymitis, or both in men vaccinated against COVID-19 [65]. They discovered that the serum levels of antisperm antibodies and antiphospholipid antibodies against cardiolipin, phosphatidylserine, annexin V, and 2-glycoprotein-1 did not differ significantly before and after vaccination. Furthermore, the levels of these autoimmune antibodies did not correlate with the sperm parameters. Their findings even suggested that COVID-19 vaccination was linked to a lower risk of orchitis, epididymitis, or both (odds ratio 0.568, 95% CI 0.497-0.649). More directly, Chillon et al [42] examined antibody concentrations in the seminal plasma of 86 men vaccinated with the new Crown pneumonia vaccine and showed that the antibody levels in seminal plasma after COVID-19 vaccination were correlated with serum antibody titers, but not with sperm parameters.

In addition, BNT162b2 and mRNA-1273 are lipid nanoparticle-formulated vaccines against SARS-CoV-2. These excipients, adjuvants, or both may act as vehicles for therapeutic content delivery while also increasing the intensity of the immune response [22]. Several studies have shown that these nanoparticles can cross biological barriers and can be deposited in reproductive organs, such as the testes, impairing sperm quality by increasing inflammation, damaging DNA structure, decreasing mitochondrial function, and inducing apoptosis [66]. Therefore, some researchers are concerned that vaccine excipients, adjuvants, or both may induce testicular damage. However, Olana et al [36] found no significant differences in the levels of reactive oxygen metabolites, electrolytes, or interleukin-6 in the seminal plasma of participants before and after the BNT162b2 vaccination [36]. Indeed, the current basic research evidence and clinical data supporting the idea that a systemic immune response after COVID-19 vaccination can lead to impaired sperm quality are insufficient.

Although semen analysis serves as the foundation for evaluating male fertility, it should be noted that it is an imperfect predictor of fertility potential. Further persuasive evidence comes from ART and artificial insemination by partner outcomes in couples after receiving the COVID-19 vaccine [57,67]. In a prospective cohort study involving 735 infertile couples, Dong et al [47] demonstrated that the quality of the embryos and the pregnancy rate in IVF treatment were unaffected by the couples’ vaccination status or vaccine type. Similarly, no differences in fertility or pregnancy outcomes were observed in the study by Orvieto et al [57]. In another multicenter prospective study of 4185 couples, Wang et al [68] found no association between COVID-19 vaccination status (eg, inactivated, adenoviral, and recombinant vaccines) and pregnancy rates in artificial insemination by partner treatment [68].

More directly, using data from an internet-based preconception cohort study, Wesselink et al [69] investigated the relationship between COVID-19 vaccination and SARS-CoV-2 infection with fertility in couples attempting spontaneous pregnancy [69]. Their results suggested that male SARS-CoV-2 infection may be associated with a short-term decline in fertility, whereas COVID-19 vaccination had no effect on fertility in either partner.

### Limitations

It is important to acknowledge that this review has a few limitations. First, data from most relevant studies were observational and lacked complete follow-up and analysis of missing data. All the included studies assessed vaccination based on self-reports or with no descriptions. Although previous influenza vaccination validation studies found 97% agreement between self-report and medical records, the conclusion may not be directly generalizable to COVID-19 vaccination, and misclassification risk may still exist [70]. Second, there is a need for more robust studies, with more precise eligibility criteria, appropriate sample sizes, and a more representative population, not focusing only on specific groups, such as those undergoing IVF. Third, despite the transient alterations in sperm parameters found in some studies, these findings were often not well supported by histological or pathophysiological data. Furthermore, the potential implications for sperm biology, including mitochondrial function, and the possible effects on the regulatory systems of epigenetic changes have received little attention.

However, given the widespread and ongoing spread of COVID-19, the available data on decreased semen quality in survivors of COVID-19 outweigh concerns about the potential negative effects of the COVID-19 vaccine on sperm parameters [71,72]. From the perspective of reproduction, vaccination against COVID-19 is preferable for severe adverse symptoms of the SARS-CoV-2 infection. Large-scale prospective cohort studies and randomized controlled trials of existing COVID-19 vaccines and newly developed vaccines are needed to further confirm this review’s conclusion.

### Conclusions

In summary, the data in this review show that the COVID-19 vaccine is safe for male reproductive health. Serious side effects of the COVID-19 vaccine are extremely rare, and men experience few problems with sperm parameters or reproductive potential after vaccination. Considering that SARS-CoV-2 infection itself may be associated with impaired fertility, vaccination could serve as a potential tool to preserve male fertility by preventing COVID-19. Therefore, vaccination should be clearly recommended for all men wishing to have children unless there are additional contraindications.

## Appendix

Multimedia Appendix 1 Filled-in PRISMA (Preferred Reporting Items for Systematic Reviews and Meta-Analyses) checklist.

Multimedia Appendix 2 Search strategy in electronic databases.

## Abbreviations

ART: assisted reproductive technology
IVF: in vitro fertilization
mRNA: messenger RNA
PRISMA: Preferred Reporting Items for Systematic Reviews and Meta-Analyses
TMSC: total motile sperm count

## Data Availability

All data generated or analyzed during this study are included in this published paper (and its supplementary information files).
