# Supplementary material for: Effects of SARS-CoV-2 Vaccines on Sperm Quality: Systematic Review
Source: JMIR Public Health Surveill. 2023 Dec 6;9:e48511. doi: 10.2196/48511 (PMC10702876; doi:10.2196/48511)
Supplement: Multimedia Appendix 2 [file publichealth_v9i1e48511_app2.docx]

**Title:** Effects of SARS-CoV-2 Vaccines on Sperm Quality: Systematic Review

Multimedia Appendices

**Multimedia Appendix 2.** Search strategy in electronic databases

Last search performed on October 1th, 2023

**Pubmed**

("COVID-19"[Mesh] OR COVID19 OR covid19 OR covid-19 OR coronavirus OR “novel coronavirus” OR “new coronavirus” OR “SARS Coronavirus 2 Infection” OR “2019 Novel Coronavirus Disease” OR “2019 Novel Coronavirus Infection” OR “SARS-CoV-2” OR “severe acute respiratory syndrome coronavirus 2” OR “COVID-19 Virus Disease” OR “COVID-19 Virus Infection” OR “Coronavirus Disease-19”) AND ("Vaccines"[Mesh] OR vaccine* OR vaccination OR "COVID-19 Vaccines"[Mesh] OR "COVID-19 Vaccines/adverse effects"[Mesh] OR “SARS-CoV-2 Vaccine*” OR “Coronavirus Disease 2019 Vaccine*” OR “2019-nCoV Vaccine*” OR “SARS Coronavirus 2 Vaccine*” OR “BNT162b2 mRNA vaccine” OR “COVID-19 mRNA Vaccine*”) AND ("Fertility"[Mesh] OR Fecundability OR Fecundity OR "Infertility, Male"[Mesh] OR "Infertility, Female"[Mesh] OR sterility OR IVF OR "Fertilization in Vitro"[Mesh] OR “reproductive function” OR ART OR “sperm” OR “spermatozoa” OR “semen analysis” OR spermatogenesis OR “semen parameter*”)

**Results :** 854 (23.10.01.)

**Scopus**

( covid19  OR  covid-19  OR  coronavirus  OR  "novel coronavirus"  OR  "new coronavirus"  OR  "COVID-19 Virus Disease"  OR  "SARS-CoV-2"  OR  "severe acute respiratory syndrome coronavirus 2" )  AND  ( vaccines  OR  vaccine*  OR  vaccination  OR  "COVID-19 Vaccines"  OR  "SARS-CoV-2 Vaccine*"  OR  "SARS Coronavirus 2 Vaccine*"  OR  "BNT162b2 mRNA vaccine"  OR  "COVID-19 mRNA Vaccine*" )  AND  ( fertility  OR  fecundability  OR  fecundity  OR  "Male Infertility"  OR  "Female Infertility"  OR  ivf  OR  "Fertilization in Vitro"  OR  "reproductive function"  OR  "ART"  OR  "semen*"  OR  "semen analysis"  OR  "spermatogenesis"  OR  " spermatozoa"  OR  "semen parameter*" )

**Results :** 2,275 (23.10.01.)

**Web Of Science**

(COVID19 OR covid-19 OR coronavirus OR “novel coronavirus” OR “new coronavirus” OR “COVID-19 Virus Disease” OR “COVID-19 Virus Infection” OR “Coronavirus Disease-19” OR “COVID-19 Pandemic*” OR “SARS Coronavirus 2 Infection” OR “2019 Novel Coronavirus Disease” OR “2019 Novel Coronavirus Infection” OR “SARS-CoV-2” OR “severe acute respiratory syndrome coronavirus 2”) AND (Vaccines OR vaccine* OR vaccination OR "COVID-19 Vaccines” OR "COVID-19 Vaccines adverse effects" OR “SARS-CoV-2 Vaccine*” OR “Coronavirus Disease 2019 Vaccine*” OR “2019-nCoV Vaccine*” OR “SARS Coronavirus 2 Vaccine*” OR “BNT162b2 mRNA vaccine” OR “COVID-19 mRNA Vaccine*”) AND (Fertility OR Fecundability OR Fecundity OR "Male Infertility” OR "Female Infertility” OR infertility OR sterility OR IVF OR "Fertilization in Vitro" OR “reproductive function” OR ART OR “sperm” OR “spermatozoa” OR “semen analysis” OR spermatogenesis OR “semen parameter*”)

**Results :** 623 (23.10.01.)

**Embase**

(COVID19 OR covid-19 OR coronavirus OR 'novel coronavirus' OR “new coronavirus' OR 'COVID-19 Virus Disease' OR 'COVID-19 Virus Infection' OR 'Coronavirus Disease-19' OR 'COVID-19 Pandemic*' OR 'SARS Coronavirus 2 Infection' OR '2019 Novel Coronavirus Disease' OR '2019 Novel Coronavirus Infection' OR 'SARS-CoV-2' OR 'severe acute respiratory syndrome coronavirus 2') AND (Vaccines OR vaccine* OR vaccination OR 'COVID-19 Vaccines' OR 'COVID-19 Vaccines adverse effects' OR 'SARS-CoV-2 Vaccine*' OR 'Coronavirus Disease 2019 Vaccine*' OR '2019-nCoV Vaccine*' OR 'SARS Coronavirus 2 Vaccine*' OR 'BNT162b2 mRNA vaccine' OR 'COVID-19 mRNA Vaccine*') AND (Fertility OR Fecundability OR Fecundity OR 'Male Infertility' OR 'Female Infertility' OR infertility OR sterility OR IVF OR 'Fertilization in Vitro' OR 'reproductive function' OR ART OR sperm OR spermatozoa OR 'semen analysis' OR spermatogenesis OR 'semen parameter*'

**Results :** 907 (23.10.01.)

**Cochrane**

#1 MeSH descriptor: [COVID-19] explode all trees

#2 (COVID19 OR covid19 OR covid-19 OR coronavirus OR “novel coronavirus” OR “new coronavirus” OR “SARS Coronavirus 2 Infection” OR “2019 Novel Coronavirus Disease” OR “2019 Novel Coronavirus Infection” OR “SARS-CoV-2” OR “severe acute respiratory syndrome coronavirus 2”):ti,ab,kw (Word variations have been searched)

#3 #1 OR #2

#4 MeSH descriptor: [Vaccines] explode all trees

#5 (vaccination OR "COVID19 Vaccines" OR “SARSCoV2 Vaccine” OR “Coronavirus Disease 2019 Vaccine” OR “2019nCoV Vaccine” OR “SARS Coronavirus 2 Vaccine” OR “BNT162b2 mRNA vaccine” OR “COVID19 mRNA Vaccine”):ti,ab,kw (Word variations have been searched)

#6 #4 OR #5

#7 MeSH descriptor: [Fertilizers] explode all trees

#8 (Fecundability OR Fecundity OR "Male Infertility" OR "Female Infertility" OR sterility OR IVF OR "Fertilization in Vitro" OR “reproductive function” OR ART OR sperm OR spermatogenesis OR spermatozoa OR “semen analysis” OR “semen parameter*”):ti,ab,kw (Word variations have been searched)

#9 #7 OR #8

#10 #3 AND #6 AND #9

**Results :** 32 (23.10.01.)
